# Technology and the clinical encounter: a qualitative study of mental health clinician and patient experiences of telemedicine

**DOI:** 10.1186/s12913-025-13764-9

**Published:** 2026-05-27

**Authors:** Emma Felman, Ian Kerridge, Mor Vered, Paul Komesaroff

**Affiliations:** 1 Monash University, Melbourne, Australia; 2 The University of Sydney, Sydney, Australia

**Keywords:** Ethical inquiry, Telehealth, Telemedicine, Psychotherapy, Methods in qualitative inquiry, Qualitative evaluation, Interpretative phenomenological analysis, Therapeutic relationship, Clinician/patient interaction

## Abstract

**Background:**

Despite the widespread use of telehealth, particularly throughout the COVID-19 pandemic, there has been little published evidence about the effect of telehealth on the therapeutic relationship and clinician and patient interaction. This study sought to identify the divergence in this mode of communication compared with in-person interaction for both clinicians and patients.

**Methods:**

An exploratory qualitative interview study of 22 mental health practitioners (registered psychologists or psychiatrists) in Australia who have treated patients using psychotherapy in-person and using telehealth, followed by a qualitative interview study of 20 patients in Australia who have undergone psychotherapy in-person and using telehealth. Using an interpretative phenomenological analysis, this paper examines participants’ perspectives on, communication with, and reactions to telehealth when providing or receiving psychotherapy treatment.

**Results:**

Both clinicians and patients described the benefits and detriments of clinical interactions using telehealth versus face-to-face. Both groups drew attention to the evident “convenience” inherent in the use of telephone or video contact, while also noting how the absence of a shared physical space fundamentally changed the conduct of private conversations with affective content. Many clinicians expressed the view that telehealth induced an underlying and intrinsic shift in the connection with the patient. Patients, likewise, reported a corresponding change in interpersonal interaction, although many experienced difficulties articulating a description of this change in relational connection.

**Conclusion:**

Patients and doctors identify both benefits and limitations of telehealth in comparison with face-to-face communication. This study has found that various factors inherent in telehealth contact affect the curative value and quality of the healthcare interaction which impacts both patient and clinician. The structure and content of the therapeutic relationship varied according to whether it occurred in a telehealth setting or in-person. These results suggest that the use of telemedicine should be tailored to the specific needs, preferences and circumstances of individual patients. The results also demonstrate that the themes identified have different implications and effects depending on the clinician, patient, and clinical situation. There are indications that telehealth overall limits or constrains the exchange of meanings in the clinic, and further research is required to explore the process by which this change occurs and its meaning.

**Supplementary Information:**

The online version contains supplementary material available at 10.1186/s12913-025-13764-9.

## Introduction

Healthcare incorporates a personal and technical service that is organised around the clinical encounter and engages with the unique biological and environmental circumstances of individual patients [1]. A goal of the interaction between healthcare providers and patients, the ‘healthcare interaction’, is to improve the flow from clinical need to patient outcome. The healthcare interaction changes with the advent of new information, infrastructures, systems, configurations, and technology. The advancement of new technologies in recent decades, such as telehealth has introduced the ability to deliver healthcare virtually.

The advent of telemedicine reflects a trajectory of innovation shaped by the continuous interplay between medical necessity and technological possibility. From early analogue communication to AI-enabled remote diagnostics, telemedicine’s evolution underscores its enduring role in improving healthcare accessibility and responsiveness. Furthermore, the nature and significance of the rapid expansion of telemedicine for the delivery of clinical care has not been fully explored.

Telehealth has emerged as a transformative modality in healthcare delivery, offering a wide array of benefits that extend across clinical, operational, and societal dimensions. As digital technologies continue to evolve, telehealth has proven especially effective in enhancing accessibility, continuity, and efficiency of care, while also supporting patient-centred approaches to health service delivery. We propose the use of the term “transactional” for many of these benefits meaning that they are elements which are concrete, measurable and defined. Transactional interactions are the formal organisational aspects of medicine which are measurable and often definitive.

One of the primary advantages of telehealth is its capacity to improve access to care, particularly for populations in rural, remote, or underserved areas. By eliminating geographical barriers, telehealth enables patients to consult specialists without the need for extensive travel, thereby reducing delays in diagnosis and treatment [2]. Studies have shown that telehealth improves service reach among Indigenous communities, patients in geographically isolated regions, and those with mobility limitations [3]. 

Continuity of care is another significant benefit, particularly in the management of chronic conditions. Telehealth facilitates ongoing patient monitoring, medication adherence support, and regular follow-up consultations, which are important for conditions such as diabetes, hypertension, and heart disease [4]. Remote patient monitoring tools and mobile health applications extend the functionality of telehealth by allowing clinicians to track vital signs, facilitate early interventions, and tailor treatment plans in real time [5]. 

In terms of cost-effectiveness, telehealth has been associated with reductions in hospital readmissions, emergency department utilisation, and unnecessary in-person consultations [6]. A systematic review found that telehealth interventions in chronic disease management can reduce healthcare costs while maintaining or improving clinical outcomes [7]. Moreover, for healthcare providers, virtual visits may optimise scheduling, reduce overhead expenses, and increase patient throughput [8]. 

From a patient satisfaction perspective, telehealth has consistently been rated highly due to its convenience, time saving, and reduced need for travel and waiting times. Many patients report positive experiences with teleconsultations, appreciating the ability to engage with healthcare providers from their own homes [9]. This convenience has been found to be particularly beneficial for caregivers, parents of young children, and individuals balancing health needs with work responsibilities [10]. 

During the COVID-19 pandemic, telehealth played a crucial role in maintaining healthcare service delivery while minimising infection risks for both patients and healthcare professionals [11]. It enabled continuity of care for non-COVID conditions, supported triage of symptomatic individuals, and facilitated mental health services during periods of heightened psychosocial stress [12]. 

Additionally, telehealth offers clinical innovation opportunities. It enables interdisciplinary collaboration through virtual case conferences, supports rapid access to second opinions, and enhances training and supervision in rural or resource-constrained settings through tele-education platforms [13]. These features contribute to more integrated, collaborative models of care that align with contemporary healthcare reform priorities. While telehealth has become an indispensable component of modern healthcare delivery, it is not without limitations. These constraints span technological, clinical, ethical, and socio-economic domains, highlighting the complexity of integrating telehealth into routine clinical practice.

One of the primary limitations concerns digital access and health equity. Telehealth requires reliable internet connectivity, digital literacy, and access to appropriate devices - resources that are unevenly distributed across populations. Studies indicate that rural, elderly, low-income, and minority communities face significant barriers to telehealth access, exacerbating existing healthcare disparities [14]. For example, rural patients are more likely to lack broadband coverage, while older adults often encounter usability challenges due to unfamiliarity with technology interfaces [15]. 

Clinical appropriateness and diagnostic limitations also pose significant concerns. Certain conditions such as those requiring physical examination, auscultation, or diagnostic testing may not be adequately assessed through virtual consultations. Telehealth may lead to diagnostic delays or reduced diagnostic accuracy in such cases [16]. While some specialties adapt well to remote modalities, others like emergency medicine or orthopaedics may be constrained by the absence of tactile and spatial assessment tools [17]. 

Technological reliability, including software glitches, video lag, or platform incompatibility, can disrupt care delivery and compromise clinical outcomes. Real-time telehealth relies on stable audio-visual connections, yet even brief interruptions can affect rapport-building, clinical judgement, and patient satisfaction [18]. Furthermore, not all telehealth platforms are designed with accessibility features such as captioning, translation, or screen reader compatibility, potentially excluding patients with disabilities or limited English proficiency [19]. 

Another limitation lies in clinician workload and workflow integration. Although telehealth is often portrayed as time-saving, clinicians frequently report increased administrative burdens, technical troubleshooting responsibilities, and workflow disruptions [9]. Additionally, clinical guidelines and reimbursement models have not always kept pace with telehealth expansion, leaving providers uncertain about standards of care and compensation for virtual services [20]. 

Lastly, therapeutic relationship-building, a cornerstone of quality care, may be altered in virtual settings. Non-verbal cues, subtle affective signals, and embodied presence are attenuated in remote encounters, potentially weakening empathy and interpersonal attunement, particularly in psychotherapy [12]. There has been limited study of the impact of telehealth on the nature and structure of the therapeutic relationship, particularly in clinical contexts like mental health care, where the communication between clinician and patient is likely to have a profound therapeutic benefit and is both verbal and non-verbal. This deficit in our understanding of the impacts of telemedicine on mental health care is likely to be particularly relevant to psychotherapeutic practices, such as psychotherapy.

“Psychotherapy” is a broad term describing a type of psychological treatment helping individuals experiencing varied mental health conditions and emotional challenges. Psychotherapy was originally developed by Sigmund Freud and Joseph Breuer in 1893 as a treatment, theory and investigative tool. There are several types of psychotherapy provided in modern psychiatric care, including cognitive behavioural therapy, interpersonal therapy, dialectical behaviour therapy, psychodynamic therapy, and supportive therapy. Therapy is typically conducted in an individual, family, couple or group setting with sessions held recurrently for 45 to 50 min. Patients and therapists are both actively involved in psychotherapy and the therapeutic trust and relationship are important for working together effectively. Psychotherapy can be recommended to deal with short term challenges for a few weeks to months or longer term (months to years) to treat long-standing complex psychiatric issues [21]. 

A key phenomenon in psychoanalytic treatment is transference - the redirection or projection of feelings, attitudes, desires or fantasies from the patient to the clinician. These emotions are often subconscious and relate to childhood thoughts and experiences. Non-verbal behaviours are particularly important in transference as they shed light on these feelings. Many non-verbal behaviours are subconscious and can represent a more accurate depiction of a patient’s attitude or emotional state [22]. It is these non-verbal behaviours which may not be conveyed through telehealth. We therefore identified and analysed the therapeutic setting of psychotherapy to focus on these “relational” aspects of the clinical interaction. A comparative analysis between telehealth and in-person psychotherapy interactions from both the clinician and patient perspectives has not previously been undertaken. This exploratory study sought to identify and explore the interpersonal benefits and limitations of telemedicine, by analysing the perspectives of psychotherapy providers and patient provided during interviews about their experiences with telehealth. An analysis of these experiences, as undertaken here, may then contribute to the landscape of telehealth and clinical communication by establishing which elements are lost in virtual communication, which then may affect clinician or patient choices to use telemedicine.

## Literature background

Existing literature has established the importance of non-verbal communication in psychotherapy [23]. Non-verbal cues including facial expressions, gestures, posture, vocal tone, eye contact, and bodily proximity are integral to establishing rapport, conveying empathy, and regulating the therapeutic alliance [24]. These forms of communication often operate below the level of conscious awareness, yet they significantly shape the interpersonal dynamics between therapist and client.

Early research in the 1980s emphasised that in emotionally charged situations, as much as 93% of communication may be transmitted through non-verbal channels, underscoring their primacy in affective expression [25]. While this specific figure has been debated, the broader consensus in psychotherapy literature recognises that non-verbal behaviours convey critical emotional information that complements or contradicts verbal content [26]. 

Within psychodynamic and humanistic traditions, non-verbal communication is seen as a pathway to unconscious material and relational attunement. Dynamic, time-based elements of non-verbal expression such as tempo and rhythm convey nuanced aspects of subjective experience that may go beyond verbal articulation [27]. Similarly, it has been proposed that micro-regulations of non-verbal interaction, such as gaze and prosody, are foundational to intersubjective coordination in the therapeutic dyad [28]. 

Empirical studies have consistently linked non-verbal synchrony with positive therapeutic outcomes. For example, movement synchrony between client and therapist predicts stronger therapeutic alliances and more favourable session ratings [29]. Likewise, non-verbal immediacy such as forward leaning and open gestures has been associated with increased client disclosure and reduced anxiety [30]. These findings suggest that the embodied dimensions of therapy significantly influence client engagement and perceived empathy.

Moreover, non-verbal communication becomes particularly important in contexts where verbal expression is constrained, such as with trauma survivors, children, or clients with neurodivergent communication profiles. In such cases, therapists’ sensitivity to non-verbal cues can facilitate emotional safety and promote attuned responses that validate the client’s experience [31]. 

Non-verbal communication is not ancillary but foundational to the therapeutic process. It enriches verbal dialogue, facilitates emotional connection, and provides a medium through which therapeutic presence and empathy are conveyed and perceived. The rise of telehealth has introduced significant changes to the nature of interpersonal communication in clinical settings, prompting a growing body of research into how these modalities affect relational dynamics between healthcare providers and patients. While telehealth enhances access and convenience, it also reshapes traditional forms of clinical interaction by altering the modalities through which communication occurs.

One major shift concerns the attenuation of non-verbal cues, such as body language, facial micro-expressions, and physical proximity, which play a critical role in the conveyance of empathy, attentiveness, and trust [24]. In face-to-face interactions, these cues contribute to the therapeutic alliance and patient satisfaction. However, in telehealth these elements may be diminished or distorted due to limited camera angles, poor resolution, or audio delays [32]. General practitioners have reported challenges in “reading” patients during video consultations, which in some cases led to less nuanced understanding of patient concerns [8]. 

Despite these challenges, studies also show that telehealth can support relational communication in particular contexts. For instance, some patients report feeling more at ease and empowered to speak openly in virtual settings, possibly due to the comfort of being in their own environment or a perceived reduction in clinical hierarchy [33]. This has been especially evident in mental health services, where video-based therapy has, for some, reduced anxiety associated with in-person consultations [34]. 

Another dimension involves technological mediation of turn-taking and conversational flow. Video latency or platform instability can disrupt synchronous communication, leading to conversational overlaps or delays in response that affect the natural rhythm of dialogue [35]. These disruptions can cause frustration and impair mutual understanding, particularly in complex discussions or sensitive disclosures. However, clinicians who receive training in telecommunication etiquette and adapt their pacing and response strategies tend to manage these barriers more effectively [36]. 

Importantly, interpersonal communication in telehealth is shaped by context, including the type of clinical relationship, the duration of the therapeutic engagement, and patient familiarity with digital communication tools. Established patient–provider relationships tend to transition more smoothly into virtual environments compared to first-time consultations, where the absence of embodied cues may hinder rapport development [37]. Additionally, cultural factors, digital literacy, and patient age can influence how communication is perceived and enacted across telehealth platforms [15]. 

The current literature illustrates that while telehealth introduces new constraints on interpersonal communication, it also opens opportunities for innovation and adaptation. Effective telehealth communication demands a heightened awareness of verbal nuance, conscious relational attunement, and the strategic use of digital affordances to maintain therapeutic quality. In order to innovate and adapt to this virtual environment, further research is required to understand the lost interpersonal communication of this modality at a deeper level. More than the technological limitations creating barriers to communication, there is a more complex interplay of lost interactions which is also taking place. These interpersonal interactions are fundamental to the therapeutic alliance in psychotherapy. Therefore, an analysis of the use of telehealth in this speciality may shed light on the complexities which lie beneath the surface when using telehealth for clinical communication.

### Methods

We conducted a qualitative study employing elements of an exploratory interpretive phenomenological analysis (IPA) [38] to help us understand how clinicians and patients providing or receiving psychotherapy make sense of their experiences with telehealth.

IPA is a qualitative research methodology rooted in phenomenology, hermeneutics, and ideography. Developed primarily by Jonathan Smith in the 1990s, IPA is designed to explore how individuals make sense of their lived experiences, particularly in contexts involving significant personal meaning or complexity [39]. Unlike purely descriptive phenomenological approaches, IPA emphasizes a *double hermeneutic* process: the participant is trying to make sense of their world, and the researcher is trying to make sense of the participant making sense [40]. 

Methodologically, IPA is most commonly applied in small, purposive samples and is well-suited to semi-structured interviews, diaries, and other first-person narrative data. The analytical process involves several iterative steps: reading and re-reading transcripts, initial noting, developing emergent themes, searching for connections across themes, and moving from individual case analysis to a cross-case synthesis [41]. While there is no rigid formula, researchers are expected to engage reflexively with both the data and their own interpretative role, acknowledging that analysis is shaped by the researcher’s preconceptions and context [42]. 

IPA has been widely used in health psychology, clinical psychology, and education, particularly in exploring experiences of illness, trauma, identity, and adaptation. For example, it has been instrumental in illuminating patient experiences of chronic pain, bereavement, and mental health conditions [43]. Its strength lies in its capacity to reveal nuanced insights into how people interpret and give meaning to complex phenomena. It is for this reason that IPA was the most suitable methodology for the research question in this study.

The methodology adopted in this study enriches existing literature on the topic by interviewing both clinicians and patients who are engaged in a similar setting. This allows for a deeper analysis of both perspectives which may be alike in many ways, but differ in some ways too.

#### Participants and setting

Clinicians (abbreviated to “DR” in the Results) were eligible to participate if they: (1) were a registered psychologist or psychiatrist; (2) provided at least 10 h per month of psychotherapy; and (3) had used telehealth technologies for psychotherapy treatment.

Patients (abbreviated to “PT” in the Results) were eligible to participate if they: (1) were over the age of 18; (2) had received psychotherapy treatment in-person (i.e., not using telehealth); and (3) had received psychotherapy treatment using telehealth.

A purposive recruitment approach was implemented using criterion sampling [44]. Potential participants were identified and recruited through personal contacts, professional organisations, and social media sites. For the recruitment of clinicians, publicly available email addresses were sourced from the website directories of two professional organisations.^1^ Email invitations were sent to psychiatrists and psychologists located in regional or urban Victoria, Australia with listed email addresses on these registries. Clinicians were not asked to invite their patients to participate, so it was unlikely that there were any connections between clinician and patient participants.

Patient participants were primarily recruited using social media. Information about the study was posted on twenty-one Facebook support groups^2^ which provide social media communities and mental health guidance for members of the public, some of which have large membership bases.

Twenty-three not-for-profit organisations^3^ assisted directly with patient participant recruitment by including the study advertisement on their social media pages, websites and e-newsletters distributed to members. Researchers’ details were provided to the potential participants, who were invited to contact us if they sought further information or wished to volunteer.

Prior to public postings, formal approval was obtained from administrators of the Facebook pages and not-for-profit organisations, who were all provided with copies of the advertisement, consent form and plain language statements. These documents described the study in detail and outlined eligibility criteria and potential risks, benefits, and requirements of participation.

The participants were relatively heterogenous, with almost equal gender representation and patient and clinician representation.

The purposive sampling strategy coupled with the lack of snowballing sampling practically eliminated potential coercion and protected confidentiality. Specific attention was paid to maintaining independence between the clinicians and patients interviewed by not asking clinician participants for assistance in recruiting their own patients for the study. All participants provided fully informed, written consent.

#### Data

Data were collected using one-on-one in-depth semi-structured interviews (see supplementary material). Consistent with the interpretive phenomenological approach, the interview schedules were flexible and non-directive, to facilitate participants speaking freely about their experiences and perspectives [45]. 

Due to the COVID-19 pandemic during the interview period, most interviews occurred online, which also facilitated recruitment across a large geographical area. The interviewees chose their preferred interview modality − 13 interviews were conducted in-person, 25 interviews were conducted by Zoom with video functionality, and 4 interviews were conducted by telephone or Zoom with audio functionality only.

All interviews were conducted in a confidential setting and all interviewees attended alone. The length, breadth and depth of interviews were determined in consultation with the interviewees, with the interviews ranging from 17 to 59 min (average clinician interview duration = 34.6 min, average patient interview duration = 27.3 min). Further information regarding the clinician participants is provided in Table 1. Further information regarding the patient participants is provided in Table 2.

Reflecting on our own experience undertaking the interviews using both modalities (in-person and via audio or video call), our sentiments mirrored those of the participants. It was far more convenient to undertake the interviews using video call. However, there was a distinct difference in the depth of interpersonal interaction which was significantly limited in the video and especially audio calls compared with an in-person discussion. Also, the experience of meeting in person, usually in the participant’s workplace, allowed for greater rapport and flexibility in communication compared with an audio or video call.

**Table 1:** Clinician participant demographics

Participant	Gender	Occupation	Interview length (m)	Interview mode
DR1	M	Psychiatrist	55	In person
DR2	M	Psychologist	34	Zoom
DR3	F	Psychologist	38	In person
DR4	M	Psychiatrist	29	In person
DR5	M	Psychiatrist	33	In person
DR6	M	Psychologist	33	Zoom
DR7	M	Psychiatrist	34	In person
DR8	M	Psychiatrist	40	Zoom
DR9	F	Psychologist	25	In person
DR10	F	Psychiatrist	39	In person
DR11	M	Psychiatrist	33	In person
DR12	F	Psychiatrist	29	In person
DR13	F	Psychiatrist	23	Zoom
DR14	F	Psychologist	38	In person
DR15	M	Psychiatrist	27	Phone
DR16	M	Psychiatrist	25	Phone
DR17	M	Psychiatrist	26	In person
DR18	M	Psychologist	59	In person
DR19	M	Psychiatrist	43	Zoom
DR20	M	Psychiatrist	44	In person
DR21	F	Psychiatrist	32	Zoom
DR22	F	Psychiatrist	23	Zoom

**Table 2:** Patient participants demographics

Participant	Gender	Interview length	Interview mode
PT1	F	22	Zoom
PT2	F	46	Zoom
PT3	M	41	Zoom
PT4	F	20	Zoom
PT5	F	29	Zoom
PT6	M	29	Zoom
PT7	F	30	Phone
PT8	M	28	Zoom
PT9	F	23	Zoom
PT10	F	37	Zoom
PT11	M	21	Zoom
PT12	M	23	Zoom
PT13	F	39	Zoom
PT14	M	22	Zoom
PT15	F	35	Zoom
PT16	F	17	Zoom
PT17	F	20	Phone
PT18	F	29	Zoom
PT19	F	17	Zoom
PT20	F	17	Zoom

Given the possibility that discussion of mental illness could exacerbate symptoms, a trauma-informed approach to the patient interviews was adopted [46] following consultation with mental health professionals. Non-threatening, non-judgmental and appreciative language and attitudes were employed, and participants were allowed to decide what and how much information to divulge. The aim of the interviews was to explore the experiences of patients undergoing psychotherapy using telehealth, so details of actual psychiatric diagnoses and treatment trajectories were not discussed unless offered by the interviewees. Patients were explicitly informed that they were not required to discuss their mental health history or diagnoses.

Topics discussed with clinician interviewees included overviews of their professional backgrounds and psychotherapy practices, thoughts and experiences relating to providing therapy via telehealth, experiences with interpersonal aspects of psychotherapy in-person and via telehealth and whether they intended to continue providing psychotherapy treatment in the future. Topics discussed with patient interviewees were informed by the clinician interview responses and included thoughts and experiences relating to therapy via telehealth and face to face, therapeutic communication, preparation before appointments, reflection following sessions, recollections of therapy, distractions using telehealth, and future preferences regarding treatment. Interviews were audio-recorded and transcribed verbatim with the assistance of Otter.AI.

#### Data analysis

All interviews were conducted by the first author (E.F), and many were also attended by another author (P.K). Reflexive thematic analysis (RTA) [47] was manually employed by the first author to identify, analyse and report themes within the data. This provided flexibility and a rich, detailed, complex evaluation. Transcripts were systematically reviewed and discussed with the other authors using the above RTA approach and important sections of text were identified and labelled. Components or fragments of ideas or experiences arising from the data were then organised into codes. Codes were iteratively reviewed for interconnectedness to see where themes should be merged or split [48]. 

RTA is a qualitative analytic method for identifying, analysing, and interpreting patterns of meaning (themes) within data. Developed by Braun and Clarke, RTA is grounded in a constructivist epistemology and emphasises the active role of the researcher in meaning-making, viewing themes as constructed rather than discovered entities [49]. Unlike more positivist approaches to thematic analysis, RTA resists notions of coding reliability or consensus and instead privileges depth of interpretation, researcher subjectivity, and theoretical flexibility [50]. 

The RTA process involves six recursive phases: (1) familiarisation with the data, (2) generating initial codes, (3) constructing themes (inferred by the authors who were informed by the prior literature as described in the Background section above), (4) reviewing themes, (5) defining and naming themes, and (6) producing the report [49]. These stages are not linear but iterative; researchers move back and forth between phases as deeper insights emerge. In this framework, coding is not treated as a mechanical or objective categorization of semantic content, but as an interpretive act where the researcher’s theoretical lens and reflexivity shape the coding process [51]. 

During the coding phase, the first author began by systematically engaging with the interview transcripts, line-by-line, identifying and labelling segments of text that are relevant to the impact of telehealth on a therapeutic interaction. An initial set of codes emerged through this analysis. The codes were then sorted into potential themes by grouping together contextual similarities. The first author (E.F.) and last author (P.K.) created codes independently. Once all transcripts had been analysed, the initial set of themes were developed. Any inconsistencies were discussed with the remaining authors and an agreement reached, after which the final list of themes were compiled by the first author who also selected relevant extracts. Data management and analysis were undertaken manually.

#### Sample size

The paradigm of theoretical saturation determined the sample size. As we coded and reviewed transcripts, the categories and patterns we identified began to repeat rather than expand. Early in the analysis, each interview contributed novel codes, nuanced perspectives, and unexpected relationships between concepts. Over time, however, we observed that the same themes were recurring with similar depth and variation, and that further interview were was merely reinforcing, rather than altering, our thematic framework. Saturation was therefore decided by consensus between the authors when additional information obtained from subsequent interviews no longer generated new understanding but, instead, continued to fit into existing categories and codes that had already been developed in the data analysis process [52]. 

Theoretical saturation refers to the point at which additional data collection no longer yields novel insights or properties relevant to the emerging theoretical categories [53]. At this stage, the categories are well developed and the interconnections among them are sufficiently robust to support theory construction.

Importantly, saturation is not synonymous with data repetition; rather, it is a marker of conceptual completeness. Saturation is achieved when further data fail to provide new theoretical insights or develop properties of the core categories [54]. This process requires simultaneous data collection and analysis, where constant comparison drives iterative refinement of theoretical constructs.

Theoretical saturation involves both the depth and breadth of conceptual development, necessitating reflexive engagement and a clear audit trail of analytical decisions [55]. It is therefore not a numerical threshold, but a methodological judgement grounded in the coherence and explanatory power of the categories.

## Results

### Demographics

All interviewees lived in regional or urban areas of Victoria, Australia. Interviews were conducted with 22 clinicians − 16 psychiatrists and 6 psychologists, with 8 female and 14 male clinicians. Interviews were conducted with 20 patients − 14 females and 6 males. Further demographics of all participants are outlined in Tables 1 and 2.

### Themes

The thematic analysis demonstrated three key themes differentiating clinical interactions through telehealth, referred to as “Convenience”, “Settings” and “Interpersonal interactions” as demonstrated in Table 3:

**Table 3:** Reflective thematic analysis

Theme	Example Codes	Description
Convenience	− “I can be at home” − “Eliminates travel” − “Feel safe” − “Easier and flexible”	The quality of reducing effort, time, or complexity in achieving a desired outcome, thereby making an action, service, or process easier or more accessible.
Settings	− “Waiting room” − “Two dimensional” − “Background”	The physical, social, or situational context in which an event, interaction, or process occurs, shaping how it is experienced and interpreted.
Interpersonal Interactions	− “Non-verbal cues” − “Eye contact” − “Body language”	The reciprocal exchange of communication, behaviours, and social cues between two or more individuals, through which relationships, understanding, and meaning are constructed.

#### Convenience

Many of the participants, both patients and clinicians, referred to “convenience” as one of the benefits of telehealth. It should be noted that while this term was used rather loosely and with various connotations, as explored below, participants generally described the impact of less physical effort and/or time being required to attend an appointment online. There was a broad assumption, which was nonetheless not universally shared, that fewer physical and/or temporal demands associated with attending a medical appointment were of benefit. Even though convenience is not strictly a relational aspect of the therapeutic interaction, it is included here given that it was mentioned by almost all participants. It is therefore an import factor in choosing whether an appointment will be held by telehealth, whether the choice is by the patient or the clinician. This choice may then result in the further thematic relational considerations explored below.

1. **Leisure time**: Many clinicians reported that the convenience of telehealth enriched their personal lives, allowing more leisure time:

*In between sessions*,* if a patient doesn’t turn up*,* I can go to the shops*,* I can go into the garden*,* I can do it from home*,* I don’t even have to show up [at work]* (DR1).

*It’s obviously convenient*,* I can work from home*,* I can work from the rooms. I flirted with the idea of one day actually having a holiday somewhere warm in winter and I can imagine that I might do part time work while I’m away having a holiday*,* so it does allow a lot of flexibility for me* (DR8).

2. **Location of telehealth**: Despite this convenience some clinicians preferred using telehealth from their workplace rather than from home:

*I needed to maintain an important boundary between a leisure environment or a domestic environment and a work environment and that the space corresponds with different internal spaces in my head* (DR1).

3. **Patient perception and safety**: Clinicians also tried to understand and respond to the needs of their patients. Generally, practitioners thought that there was a patient preference for telehealth:

*It was actually much more convenient for the patients to come for telehealth rather than traipsing an hour each way to come to a session*,* so I’ve decided to make my practice entirely telehealth* (DR3).

The related concept of safety was also mentioned by practitioners, given the vulnerable thoughts and feelings of people undergoing psychotherapy treatment:

It’s very convenient and less stressful for both parties. I think the best thing about it is that both parties feel safe…I think patients love the idea even more than psychiatrists and psychologists do (DR4).

However, some practitioners were unsure of their patients’ preferences and reflected on the predominant inclination:

*I guess I would like to know*,* I guess I could ask my own clients* (DR9).

There were some patients who preferred in-person interactions and expressed concern about the continued use of telehealth:

*I’ve been worried that once COVID is completely over*,* we’re not going back to face-to-face* (PT19).

Some patients also mentioned a preference for in-person appointments despite the convenience of telehealth:

*I think it’s a wonderful means of communication with your medical practitioner*,* whether it is a doctor or counsellor or whatever*,* whoever. But I don’t think that it applies or satisfies the needs of everyone. Definitely not mine…I personally like the direct contact*,* person to person being in the same room* (PT12).

4. **Urban versus rural**: Being located outside an urban area was also a factor which highlighted the benefit of convenience.

*Because we worked up in Albury Wodonga for a long time*,* before we moved to Melbourne*,* a lot of these patients stayed with me and would come down to Melbourne and drive sort of a seven or eight hour return trip just to come see me or they catch the train*,* and it was a whole palaver. So*,* telehealth has become something that’s really convenient for those people. Not everybody loves it but most people*,* in fact*,* have found it fantastic and they’ve preferred to do that rather than come to Melbourne* (DR8).

5. **Flexiblity**: Allowing flexibility around work commitments also favoured telehealth for convenience reasons:

*Even patients in the city who would have ordinarily driven to an appointment*,* spent an hour in traffic and an hour going back in traffic and had to park and all that sort of thing and usually taken half a day or a day off work. Now they find it easier*,* they can step into a private office at work*,* or they can go and sit in the car. They can do a half hour appointment without completely disrupting the day’s work* (DR8).

6. **Maintaining therapeutic relationship at a distance**: A patient or clinician relocating would usually prompt a patient to find a closer therapist. Telehealth now allowed the therapeutic relationship to continue:

*One o my patients lives in Geelong now*,* but it’s fine with telehealth*,* far better than changing therapists just because geography is in the way*,* and you can continue this positive constructive relationship* (DR9).

7. **Accessibility**: Almost all patients reported telehealth to be a more convenient and accessible way to regularly speak with their mental health clinician of choice:

*The preference is always telehealth because it’s just so much more convenient for me* (PT4).

*I don’t have to travel; I find just getting organised and constructing my day around going out somewhere and…driving for an appointment is really difficult so it’s really convenient to do that from home* (PT1).

#### Settings

Conventionally, there is a historical insistence that psychological therapies are delivered in-person [56]. The central premise to this argument is that the effectiveness of these interventions is reliant upon the development of a high-quality therapeutic alliance between clinician and patient. The use of the telephone or internet video technologies invariably eliminates the physical presence of clinician and patient. It is possible that an effective alliance is reduced in the absence of this physical presence [6]. 

“Settings” in this study refers to the physical presence of the clinician and patient, as well as the physical scene of the appointment. The notion of the setting, or physicality in terms of shared space, of the appointment was frequently mentioned by both patients and clinicians as an important factor in the interaction.

Both patients and doctors considered the setting to play an important role in facilitating the clinical dialogue and supporting insights and understanding about difficult psychological issues. Clinicians were focused on the layout of the physical environment while the patient focused on the experience of the setting.

1. **The waiting room**: The relevance of the waiting room was raised by some clinicians:

*The waiting room is quite an important place…we generally think of the area of work as the consulting room but the waiting room and the period before the consultation and the period after the consultation are actually quite important* (DR1).

The physical waiting area was reported to be potentially clinically relevant to the preparation before an appointment for the patient:

*I wanted to say something about the waiting room and the period before and after. That doesn’t exist to the same extent in telehealth*,* and it was a patient who pointed this out to me. He said…when I’m in the waiting room*,* I’m thinking about things…There’s a period of having an idea and a period of frustration or anxiety in the waiting room* (DR1).

*All those constitute material for work and the disappearance of those through just clicking on a Zoom link is actually quite a sin* (DR1).

On the other hand, telehealth offered increased privacy by eliminating potential contact between patients in the waiting area:

*You don’t have that horrible feeling when you’re sitting in a waiting room*,* and you look at the person and wonder why they’re here or you see someone coming out and the smells and the receptionist and all that vibe* (DR6).

2. **Detachment**: Many practitioners mentioned the sense of feeling separated or detached from the interaction using telehealth:

*There is a two-dimensional sense of each other’s space and that can feel a bit abstract or alienating at times* (DR6).

*Just having that felt sense of the body and space…which is different to being in a room of my own. Being in [the patient’s] room as opposed to us sharing a space and the kind of automatic connection that brings* (DR2).

This was linked to the idea of conveying warmth or energy during a clinical interaction:

*One of the main things that comes to my mind in the room is that it is an embodied experience…and you’ve got not just mind communicating with mind but body with body* (DR6).

*It is more of a sense of aliveness sometimes in the room so I can feel ways there’s a numbing sometimes online…so that affects the actual experience* (DR6).

The physical consultation room was also mentioned as representing a psychological delineation of the interaction:

*You don’t want to have to deal with the patient outside the room*,* nor does the patient necessarily want to deal with you. The room is actually exclusive* (DR3).

*So you might actually see more subtle things than you realise that may contribute to the effect in the room* (DR3).

Patients also reported feeling disconnected from the energy or warmth “in the room”:

*I think the body energy is not the same as if I was there in the room…it feels more intimate to me when I am there than when I’m far away and I see them through a screen* (PT12).

*I just feel when you are talking about things that are quite personal or emotional it is important to have that human connection being in the room with them. It’s just a better feeling than just a screen. Just being able to see their reactions* (PT20).

*Telehealth just doesn’t quite have the same feel for me as being in the same room. I just find being in the same room with a psychologist I’m forced to open up a lot more and that can be therapeutic in a lot of senses* (PT3).

*Even with the best clinician or the best interviewer in a telehealth environment there’s a certain artificiality that isn’t the case with face-to-face consultations in the same room. I think there is perhaps always a little bit of strain (PT6).*

Mirroring the view of the clinicians, patients described the psychological delineation of the consultation room:

*Once I left the room and closed the door my thoughts were compartmentalised…the room itself is a safe space. It can be harder to get myself into the headspace having to do that in my home* (PT10).

*At home it feels different. It feels almost like a bit of a conversation as opposed to a clinician interaction* (PT17).

3. **Comfort**: However, clinicians reported feeling more comfortable when speaking on telehealth and noticed the increased comfort of their own patients:

*I could have moccasins on*,* and track pants and my clients wouldn’t know*,* so there’s a comfort and flexibility between clients* (DR6).

*I noticed some of my existing clients who are suffering from anxiety were more relaxed in telehealth*,* like they were able to sit on their bed with their dog on their lap stroking their dog* (DR6).

Similarly, patients described a sense of control in telehealth:

*I prefer the telehealth appointments because I can control my environment to a degree here* (PT2).

4. **Patient perception**: Clinicians mentioned the connection between the physical space and how they are portrayed to the patient:

*I’m looking for a sense that I am not positioning myself as an expert. I’m someone who is here as a kind of reflective observer and helping the client to feel comfortable in that way*,* letting them know that it’s a non-judgmental space* (DR2).

*I’m not inviting them into my home or my consulting room*,* they’re inviting me into their home*,* and I think that changes some aspects and not necessarily for better or for worse but it’s just different* (DR5).

*I focus on giving [the patients] the sense of care through my communication at all times. I’m not walking them out of the therapy room to the front door to say take care*,* get some rest*,* look after yourself tonight but I still say that on the screen* (DR6).

*Personally*,* I think with mental health it is far more important to be one-on-one* (PT17).

5. **Physicality**: Of note, the physical space of the patient had an impact on their thoughts and feelings in the therapy session. The brightness of the room was specifically mentioned:

*I try to keep a very clear head. I try to prepare the room so that it’s not going to be too bright* (PT2).

*There’s actually less distractions being in telehealth than there are having to be in an environment that you might not necessarily know especially if you’re new to that place. That can be quite distracting*,* you know*,* the smells of the room can be distracting*,* the light*,* the brightness and everything like that whereas when you are in your own place you can control the environment a lot more so less distractions* (PT4).

Patients noticed the décor and furnishing of their therapists’ rooms:

*When you are in a therapist’s office the way that they have decorated can be kind of distracting. I know one of them had a bright green wall that they sat in front of and after an hour it was really hard to look at the wall and it was distracting and hurt your eyes* (PT9).

*They are usually in an environment that is quite friendly like how they put a bit of effort into having nice furniture and things* (PT20).

#### Interpersonal interactions

Interpersonal interactions in telemedicine are shaped by both the opportunities and constraints of technology-mediated communication. While virtual consultations can preserve many aspects of rapport-building, such as active listening, empathy, and clear verbal communication, they can limit access to subtle non-verbal cues like body language, touch, and environmental context. Clinicians may need to rely more heavily on tone of voice, facial expressions, and deliberate verbal affirmations to convey attentiveness and compassion. Participants reported a difference in perception of non-verbal behaviours of the other person within the clinical interaction between telehealth and in-person therapy sessions.

1. **Non-verbal communication**: Non-verbal communication was reported to be more difficult to convey and read using telehealth:

*Reading the non-verbal cues is different. The level of connection and intimacy is different* (DR5).

*You’re not getting the full body*,* so you don’t get to read the whole non-verbal cues…there’s stuff you miss because you’re not seeing somebody’s whole body* (DR9).

*The relationship part is easier in the room*,* and I find it much harder online…in the room with a patient I can have a real human being* (DR10).

2. **Eye contact**: Clinicians focused on the difference in eye contact with their patients:

*The thing that kind of struck me most at first was not being able to make direct eye contact*,* that I kind of struggled with looking at a screen and having [the patient’s] eyes looking somewhere else not directly into mine* (DR2).

*On Zoom the amount of time we’re trying to look at each other and there’s something about the electronic medium I found it extremely exhausting* (DR6).

*I’m just a talking head with eyes that [the patients] don’t know…so I’ve really got to use my eyes much more* (DR12).

3. **Body language**: Patients described a change in body language and body language perceptibility on telehealth:

*There’s absolutely no body language or anything*,* it’s just voice and I really feel that a lot is lost even like when you’re doing telehealth even the pauses don’t have the same meaning* (PT8).

*A lot of the anxiety around telehealth is due to the fact that when you are not in person it is very difficult to read body language. You know*,* like tone and everything. It’s just much harder to build a rapport* (PT8).

*I think the communication would have been better just by being one-on-one in the room*,* it would have been more spontaneous*,* the body language would have been there. I think [my therapist] would have preferred to have seen me face to face as well because part of her job is not only to listen to what I’m saying but to notice my whole body language and presentation*,* how I’m holding up every session - how I dress*,* groom*,* my own body language and she can assess that better face to face in the consultation suite. So [telehealth] is kind of like a good but not great communication* (PT3).

*In Zoom you really just see them up close. When you are in the room with someone you kind of sit away from them*,* you can see their whole body you can see the way they are sitting*,* their body language* (PT20).

*I miss out a lot on the phone – the body language*,* the facial motions. All the cues you normally use when you are talking to somebody* (PT19).

*With face-to-face communication you gain a lot more because you’ve got facial expressions and body language and tone of voice*,* which you miss out*,* especially in email* (PT1).

## Discussion

This study shows that both clinicians and patients have an ambivalent attitude towards the use of telehealth in therapy sessions, with strengths and weaknesses recognised by both groups. Most of the participants explored considerations which are subjective and more challenging to measure and define, such as interpersonal communication between practitioner and patient and the setting of the interaction. The results demonstrate that there are also considerations which are transactional in nature, such as convenience, which can be objectively measured in time and effort.

### Transactional considerations

The main argument in support of telehealth related to what was commonly referred to as “convenience”, by which most people appeared to mean that less physical effort was required to attend a session, causing less disruption to daily life. Almost all participants, in both the patient and clinician groups, referred to the convenience of telehealth. While the meaning of the term “convenience” seems to be clear and uncomplicated, the concept of convenience in reality is not straightforward. This is explored further in our paper *Reflections on the cloak of convenience* [57]. 

From a practitioner perspective, reducing or eliminating the need for a separate place of work can decrease the associated costs of maintaining consulting rooms, staff, childcare and car or public transport travel. Working from home can also increase work capacity in lieu of travel time. For the patient, the flexibility of speaking with a clinician using a phone or computer was reported to increase the likelihood of feeling safe and being open and honest in discussions, as well as minimising disruption to employment commitments and childcare responsibilities. However, despite the obvious and compelling nature of these arguments, convenience was not seen as completely straightforward, and indeed was strongly tempered by other considerations, which were frequently stated in relation to the other two themes of “settings” and “interpersonal communication”.

### Relational considerations

Many participants described the impact of sharing a room with their patient or treating therapist and described how this impacted on their thoughts and feelings. Some patients mentioned it briefly without attributing much relevance to their own environment. Others highlighted the importance of sharing a clinical space with their therapist. Most of the clinicians, particularly the psychiatrists, reported a preference for in-person face-to-face interactions with their patients. The clinicians referred to the importance of a shared physical space both for treatment progression and developing and maintaining a rapport with their patients.

Rapport is influenced by three non-verbal behaviour elements: attentiveness, positivity-negativity, and coordination [58]. Attentiveness refers to an individual’s capability for focusing attention on the interaction occurring between the patient and clinician in the present time. If a patient feels that their therapist is distracted or uninterested this undermines rapport. A clinician can demonstrate interest in the patient with non-verbal behaviours such as a making eye contact and nodding. Positivity-negativity refers to how interacting individuals are responding to each other. Is there mutual commitment visible with smiling, laughing, and using “open” body language? Or do the non-verbal cues display indifference or hostility through creating physical distance? Coordination refers to the similarity in non-verbal behaviour of the clinician and patient. For example, making eye contact at the same time, returning a smile or mirroring body language. These subtleties extend beyond the theoretical and practical confines of convenience and can have a significant impact on the therapeutic interaction.

The importance of the environment and context of the therapeutic interaction was reflected in the data in this study. Clinicians mentioned the unfamiliarity of speaking with a patient on telehealth while the patient was sitting in their own home or workplace. Patients also spoke about the impression of brightness, décor, and furniture on their therapy session.

In some ways our results are consistent with other research on the effect of room design on clinical outcomes. For example, the presence of a window in a hospital room has been found to result in shorter preoperative stays, fewer analgesic doses and fewer minor postoperative complications for patients recovering from gallbladder surgery [59]. Patients in intensive care units without windows had higher instances of delirium than patients in rooms with windows [60]. Higher satisfaction of window view has also been found to significantly decrease analgesic usage, perceived pain and pain severity for women who had undergone caesarean Sect [61]. The value of person-window transactions is related to the patient developing a “perceptual and cognitive link with the external environment” [62]. Windows provide natural light, scenes of nature and a soothing distraction which is an asset to the therapeutic process.

Examining the influence of the total environment, rather than isolating individual variables, is another method of determining the influence of the physical environment on patient experience. Previous research on healthcare environments has suggested that patients respond positively to natural light, positive distractions such as paintings, artwork and reading materials, pleasant furnishings, plants, and warmth [63]. It has also been found that perceived quality of care and comfort level is significantly greater for waiting rooms that are nicely furnished, light, contain artwork and are warm compared with waiting areas that are dark, outdated and poorly furnished [64]. 

Importantly, many participants noted not only the ways in which telehealth impacted therapy through changes to the “settings” of clinical interactions, but through its impact on interpersonal communication.

Communication is a process in which people verbally or non-verbally share information and ideas. Non-verbal communication is a silent form of communicating with a person without using any form of speech. It is often used to express a thought or demonstrate a message in a more interesting and emotive way. Types of non-verbal communication include paralanguage, body movement, facial expressions, eye messages, appearance, clothing, body adornment, space and distance, touch, time, smell, and manners [65]. 

Nuanced visual information was explored by the participants in this study by describing behaviours such as eye contact, physical expression, posture, and voice. The basic principles of non-verbal behaviour are often categorised as proxemics (physical distance between people), kinesics (body movements) and paralanguage (non-verbal elements of speech) [66]. These characteristics are collectively referred to as “interpersonal interactions”. Research shows that non-verbal behaviours of the clinician greatly impact the dialogue in psychotherapy [37]. Just as the therapist is observing the patient, the patient is also observing the therapist. This is reflected in the results of this study – both patients and clinicians noticed and described elements of interpersonal behaviour. Non-verbal behaviour can play a significant role in establishing the therapeutic alliance in any patient-physician interaction. Psychotherapy particularly emphasises the importance of formation of rapport between the patient and therapist. Rapport is considered essential to continue to build a strong therapeutic alliance to work towards mutual goals [37]. 

During a clinical consultation, there are several observable non-verbal behaviours mentioned by the participants here that can produce information about the patient. Some of these were mentioned by the clinician participants in this study. For example, where the patient chooses to sit, posture during the interview, whether eye contact is maintained and how the patient reacts to interpretations beyond verbal acknowledgement. Over time, as with any interpersonal interaction and relationship, the clinician becomes attuned to the patient’s baseline appearance, attitude, and behaviour.

Non-verbal behaviour is interpreted within context, which is the primary reason the theme of “interpersonal communication” is linked to the theme of “settings” in these results. It is important for clinicians to notice these behavioural cues but also to draw appropriate interpretations from them. These non-verbal cues cannot be interpreted in a vacuum. There is no single behaviour or gesture which has the same meaning in every conceivable context. There are multiple layers to non-verbal behaviours to consider [67]. First, a clinician considers the environment in which an interaction is taking place. For example, a patient shivering during an initial consultation may be interpreted as apprehension or it might simply indicate an office temperature being too cold for comfort. Second, a clinician considers an individual’s typical presentation during an examination. Some people are naturally more expressive in terms of general animation, gestures and affect. Others may carefully control and modulate their feelings. Third, non-verbal behaviours are considered globally rather than centred on the minutiae. Instead of focusing on any single gesture, it is more effective and useful to accurately interpret several behaviours that occur simultaneously. Finally, a clinician reflects on the interaction occurring with the patient in real time – the clinician’s own non-verbal actions may in turn affect a patient’s behaviour, and vice versa.

The themes which have emerged in this study extend beyond the existing limitations identified by existing literature about telehealth which are explored in the *Introduction* section above. This study suggested that there are deficiencies in communication when using telehealth which are nuanced and essential, and may not be able to be resolved with more advanced technology. There are fundamental concealed differences created by the technology “intervention” which are important, but have traditionally been difficult to identify and articulate. This study demonstrates that these subtle, and not-so-subtle differences impact upon both patient and clinician in a therapeutic encounter in unexpected ways.

### Implications for patient care

The somewhat surprising vicissitudes of the concept of convenience discussed above apply remarkably appropriately to telehealth. The convenience of telehealth (in the narrow sense) is undoubtedly beneficial and almost all participants mentioned this advantage when exploring their thoughts and feelings about telehealth. Despite the very positive response to the convenience of telehealth, the value of accessibility is not the only weight in the scale of therapeutic quality. The impact of the altered interpersonal communication, combined with the change of setting, weakens the interaction between the clinician and patient.

This study shows that telehealth conveys significant benefits, which may often come with a price. The benefits related to the increased accessibility it offers to people who may not otherwise be able to avail themselves of medical care, because of physical or social constraints they may face. In some circumstances, telehealth is seen also to support communication where it might otherwise have been limited or obstructed in the face-to-face setting. However, for the majority of participants, it appeared that clinical communication was less free and open, and so less fecund, in the telehealth context than when they were face to face. In other words, the technical framework imposed by telehealth was considered to penetrate to the actual content of the transactions that ensued.

This means that the implications need to be carefully considered: while telehealth may increase the availability of care and the frequency of encounters, it may also shape the encounters in important ways, possibly limiting the meanings that are transacted or even, in some cases, extending them. Which happens to be the case in a particular setting needs to be carefully considered by the participants. It is therefore possible that telehealth and face to face consultations have different optimal clinical applications, although at the present time insufficient evidence is available to guide such a distinction.

The ultimate objective of any therapeutic interaction is to improve the patient’s health and medical care. Clinical practice brings together the expertise and acumen of the clinician with the values, perspectives and treatment preferences of the patient in order to make sense of, and treat, illness. It is proposed that healthcare interactions consist of *transactional* elements and *relational* elements. Transactional interactions are the formal organisational aspects of medicine which are measurable and often definitive. Convenience as a notion is fundamentally transactional – the time and effort saved can be defined and measured. Relational interactions refer to the non-technical or informal elements of healthcare which are identifiable but often difficult to specifically measure, such as setting and interpersonal communication.

The patients and clinicians involved in this study did not discuss, or even mention, the details of diagnoses, treatment methods or disease progression, so-called instrumental elements. The open-ended questions allowed all participants the opportunity to raise any matter they considered relevant to telehealth differences or preferences. However, the interviewees chose to explore the impact of their environment and non-instrumental aspects of the interaction. The specific exploration of these “relational” elements of care implicitly illustrates the importance of interpersonal factors within the clinical interaction.

## Conclusions

This exploratory qualitative interview study examines non-transactional aspects of clinical interactions and relational factors for patients and clinicians in telehealth. The semi-structured and in-depth nature of our interviews facilitated unanticipated findings to emerge. While there is reduced effort and time involved with telehealth, the results of this study demonstrate the importance of the relational elements such as physical setting and conveying non-verbal behaviours which were found to be vital to the interaction, and appear to outweigh the transactional benefit of time and effort saving. Despite the benefit of convenience, the change of physical presence and diminishment of interpersonal communication on telehealth means that this technology modality modifies both the structure and content of the clinical interaction. There are indications that telehealth overall limits or constrains the exchange of meanings in the clinic, further research is required to explore the process by which this change occurs and its meaning.

The primary limitation in this study is the specific clinical treatment explored here – psychotherapy provided or received using telehealth. Physical examinations are generally not required for psychotherapy [29]. However, the focus on clinical communication in terms of “settings” and “interpersonal communication” is not specific to psychotherapy. Therefore, the results are likely to be generalisable to all clinical communication, although there may be other considerations that arise in non-psychiatric clinical interactions, such as the importance of somatic or sensory experiences and the performance of a physical examination where were not explored here.

Even though the sample size was modest, theoretical saturation was achieved. Almost three times more psychiatrists than psychologists participated in the study. Limited quantitative data were collected so there was no differentiation between participants and results based on age, gender, location, diagnoses, and types of psychotherapy.

It is worth noting that there are constant advancements in the area of technology-mediated communication which might be beneficial within the clinical space. At the time of writing there are virtual waiting rooms, three dimensional platforms and metaverse options in early use or development which could be implemented to address the telehealth deficiencies identified in this project.

Future research on quality of healthcare interactions using telehealth could focus on developing an instrument that comprehensively addresses the overall technology, is sufficiently brief to use as a practical tool for patients and practitioners in a clinical setting with demonstrated reliability. Isolating specific aspects of telehealth to control and explore would enhance results on the impact of these factors on the interaction. There could also be an investigation of the treatment outcomes for patients treated via telehealth compared with in-person, as well as exploring the impact of telehealth for clinicians and patients in other specialities. Given the nuanced limitations identified in this study with telemedicine, and the importance of communication in the provision of psychotherapy, it would be particularly interesting to assess whether patient outcomes with psychotherapy differ with altered treatment modalities. In relation to communication using digital technologies such as telehealth, future research could focus on aspects of the technology which may improve the deficiencies outlined in this study.

## Supplementary Information

Below is the link to the electronic supplementary material.

Supplementary Material 1

Supplementary Material 2

## Data Availability

The data generated and analysed for this study are not available for participant confidentiality reasons. Reasonable requests for further information may be directed to the corresponding author.

## Footnotes

1 The Psychotherapy and Counselling Federation of Australia and the National Association of Practising Psychiatrists.

2 These included: *A Group for ADHD Women*, *Endometriosis Victoria Support Group*, *Multiple Myeloma Support Groups Australia*, *Melbourne Pregnancy Birth and Beyond Support Group*, *Complex PTSD Support Group Australia*, *Domestic Violence Library*, *Migraine and Headache Support Group*, *The Australian Narcissistic Support and Recovery Group*, *Fibromyalgia Support and Advice Australia*, *Gastric Sleeve Support Group Australia Wide*, *Chronic Pain Support – Australia*, *Chronic Fatigue Syndrome Support Group Victoria*, *Melbourne Pandemic Support Group*, *Grief Support Group Australia*, *Disability Inclusive Support Services Network Australia*, *Anxiety and Depression Support Group Australia*, *Ectopic and Miscarriage Support Group Australia*, *Sydney Disability Support Group*, *Anxiety Support Melbourne*, *Eating Disorder Support Group Australia*, *PTSD Research Group Australia* and *Cancer Support Group Australia*.

3 These included: ADHD Australia, ADHD Foundation, Gidget Foundation, Fearless, BEING, SANE, GROW, Mental Health Foundation, Pain Australia, Blue Space, Anxiety Disorders Association, Migraine Australia, Patient Advocates Australia, Australian Patient Association, PANDA, Chronic Pain Australia, Pain Management Australia, Mensline, Anxiety Resource Centre Victoria, Eating Disorder Council (part of the Butterfly Foundation), Blue Knot, Independent Mental Health Advocacy, Monash University Counselling and MindSpot (part of the eCentre Clinic).

## Declarations

### Data security and privacy

Given the sensitive nature of the research, protecting the privacy of participants was of particular importance. All documents (including audio-recorded interview files) were stored electronically on encrypted password-protected cloud-based drives using software approved by Monash University. Only signed consent forms contained the name and signature of participants. No other documents contained identifying information. Dates of birth and residential addresses were not collected. Participants were assigned a unique code which was used to identify interview recordings and transcripts.

### Participant reimbursement

Participants did not receive reimbursement or other compensation for assisting with the study.
