# Supplementary material for: Technology and the clinical encounter: a qualitative study of mental health clinician and patient experiences of telemedicine
Source: BMC Health Serv Res. 2026 May 27;26:757. doi: 10.1186/s12913-025-13764-9 (PMC13214332; doi:10.1186/s12913-025-13764-9)
Supplement: Supplementary file 1 — Supplementary Material 1 [file 12913_2025_13764_MOESM1_ESM.pdf]

SEMI-STRUCTURED INTERVIEW GUIDE

Project ID: 32182
Project title: Patient-centric considerations for the implementation of artificial intelligence in healthcare
Group 1 - Psychiatrists/psychologists >10 hours psychotherapy per month

Hello, my name is Emma. Thank you for agreeing to be interviewed as part of this study.

As you know from the information and consent form, the first part of this study is about talking with several psychiatrists/psychotherapists about their thoughts and experiences around delivering psychotherapy via telehealth. By saying telehealth or telemedicine we mean pre-arranged appointments with patients which take place either via video such as Zoom or over the phone for at least 30 minutes.

There are no right or wrong answers. We are interested in hearing about your experiences, thoughts and opinions.

To start with, can you tell me about your professional background and the kind of work that you do?

[Possible prompts: How long have you been treating patients using psychotherapy?]

To start with, can you tell me about your psychotherapy practice.

[Possible prompts: How much of your practice per week/month involves psychotherapy? What are the reasons you would usually recommend psychotherapy? What are the general conditions treated with psychotherapy? Are there any specific conditions which you mainly treat here? How long do the sessions go for? What are the general objectives/goals of psychotherapy? What are you hoping to achieve when a patient undertakes psychotherapy? How often are the sessions recommended? How many sessions would a patient usually have?]

Now I am going to ask you a range of questions about your experiences with telehealth in your psychotherapy practice.

Can you tell me about your thoughts and experiences relating to telehealth in your clinical psychotherapy practice?

Can you tell me your thoughts about the good and bad things about telehealth in your clinical psychotherapy practice?

Now we are going to discuss what has been termed the "informal" aspects of patient care in your psychotherapy practice. The formal or technical aspects of healthcare might include measurable identifiable aspects of the interactions such as treatment goals, treatment outcomes, severity of disorder or disease. What we call the "informal" or "relational" aspects here refer to the

non-measurable aspects such as communication, interpersonal dynamics, behaviour, rapport with the patient, etc.

Would you agree with this description of "informal" elements of patient care? Would there be anything you would like to add to this definition?

Could you tell me about your experience with these interpersonal aspects of psychotherapy in your practice?

[Possible prompts: Are these interpersonal elements important in psychotherapy? Describe the interpersonal objectives in psychotherapy?]

What are your experiences with these relational or interpersonal elements when delivering psychotherapy <u>face-to-face</u>? If you think it may be helpful to describe some actual cases please go ahead and do so.

What are some of your experiences with these relational elements when delivering psychotherapy via <u>telehealth</u>? If you think it may be helpful to describe some actual cases please go ahead and do so.

Can you elaborate on what you think are the differences between face-to-face and telehealth psychotherapy, specifically in relation to these relational aspects?

Do you intend to continue to deliver psychotherapy via telehealth as frequently as you have for the past 2 years?

[Possible prompt: What proportion of your psychotherapy practice has used telehealth in the past fortnight?]

Thanks for your time. Is there anything else you would like to add that has not been covered?
