# Supplementary material for: Technology and the clinical encounter: a qualitative study of mental health clinician and patient experiences of telemedicine
Source: BMC Health Serv Res. 2026 May 27;26:757. doi: 10.1186/s12913-025-13764-9 (PMC13214332; doi:10.1186/s12913-025-13764-9)
Supplement: Supplementary file 2 — Supplementary Material 2 [file 12913_2025_13764_MOESM2_ESM.pdf]

SEMI-STRUCTURED INTERVIEW GUIDE

Project ID: 32182
Project title: Patient-centric considerations for the implementation of artificial intelligence in healthcare
Group 2 - Patients who have had therapy in person and via telehealth

Introduction

Hello, my name is Emma. Thank you for agreeing to be interviewed as part of this study.

As you know from the information and consent form, this part of this study is about talking with people about their thoughts and experiences around having therapy via telehealth. By saying telehealth or telemedicine we mean pre-arranged appointments with your therapist which take place either via video such as Zoom or over the phone for at least 30 minutes.

There are no right or wrong answers. We are interested in hearing about your experiences, thoughts and opinions.

Background

To start with, can you tell me a little about yourself?

How long have you been having therapy and how often?

Generally, when do you have appointments with your therapist in person and when have the appointments been on video or phone?

General thoughts about telehealth

Now I am going to ask you a range of questions about your experiences with telehealth with your psychiatrist or psychologist.

Can you tell me about your thoughts and experiences relating to having therapy via telehealth?

What do you think are the good things about having therapy via telehealth?

What do you think are the bad things about having therapy via telehealth?

Communication

How do you find communicating with your therapist via telehealth?

Do you feel that you were able to establish a connection or rapport with your psychiatrist or psychologist when speaking via telehealth?

Do you feel understood by your therapist when speaking via telehealth?

Do you feel that you made progress with your treatment when speaking via telehealth?

Preparation, reflection

When having an appointment in person there might be a process involved with the preparation and reflection for that visit. When I say preparation I mean your routine before the appointment - you might get ready, travel to the therapist's rooms by car or public transport and wait in the waiting room. When I say reflection I mean that after the visit you would travel back to your home or work and during that time you might think about the topics that were discussed in the appointment. What do you think about or do before and after the face-to-face therapy appointment?

How does this compare with the preparation and reflection for the telehealth appointments?

Memory

When having therapy via telehealth, what place did you choose to have the discussion? (eg office, bedroom, car)

When you speak with your therapist in person do you feel any difference being in their rooms physically in the same space as them, compared with speaking via video or phone in your own space?

When you think about the therapy sessions that have been face-to-face versus via telehealth, do you remember them differently or recall the discussions differently? How so?

Distractions

When you speak with your therapist face-to-face you are present, in the room, and focusing on what is happening. When you speak on telehealth you are in [location mentioned above], so do you feel more distracted by your surroundings such as phone, email, other people, when in your own environment?

Future

Do you intend to continue having therapy via telehealth? Why?

Thanks for your time. Is there anything else you would like to add that has not been covered?
